# Validity of clinical disease activity index (CDAI) to evaluate the disease activity of rheumatoid arthritis patients in Sri Lanka: A prospective follow up study based on newly diagnosed patients

**DOI:** 10.1371/journal.pone.0278285

**Published:** 2022-11-29

**Authors:** Keerthie Dissanayake, Chandrika Jayasinghe, Priyani Wanigasekara, Jayampathy Dissanayake, Ajith Sominanda

**Affiliations:** 1 Department of Anatomy, Faculty of Medicine, University of Peradeniya, Peradeniya, Sri Lanka; 2 Department of Medicine, Faculty of Medicine, University of Peradeniya, Peradeniya, Sri Lanka; 3 Teaching Hospital, Peradeniya, Sri Lanka; 4 Rehabilitation Hospital, Digana, Sri Lanka; 5 Department of Basic Medical Science, College of Medicine, Qatar University, Doha, Qatar; Kansai Medical University: Kansai Ika Daigaku, Institute of Biomedical Science, JAPAN

## Abstract

Routine use of the Disease Activity Score-28 (DAS28) to assess the disease activity in rheumatoid arthritis (RA) is limited due to its dependency on laboratory investigations and the complex calculations involved. In contrast, the clinical disease activity index (CDAI) is simple to calculate, which makes the "treat to target" strategy for the management of RA more practical. We aimed to assess the validity of CDAI compared to DAS28 in RA patients in Sri Lanka. A total of 103 newly diagnosed RA patients were recruited, and their disease activity was calculated using DAS 28 and CDAI during the first visit to the clinic (0 months) and re-assessed at 4 and 9 months of follow-up visits. The validity of the CDAI, compared to DAS 28, was evaluated. Patients had a female preponderance (6:1) and a short symptom duration (mean = 6.33 months). Internal consistency reliability of CDAI, as assessed by Cronbach’s α test, was 0.868. Convergent validity was assessed by correlation and Kappa statistics. Strong positive correlations were observed between CDAI and DAS 28 at the baseline (0 months), 4 and 9 months of evaluation (Spearman’s r = 0.935, 0.935, 0.910, respectively). Moderate-good inter-rater agreements between the DAS-28 and CDAI were observed (Weighted kappa of 0.660, 0.519, and 0.741 at 0, 4, and 9 months respectively). Discriminant validity, as assessed by ROC curves at 0, 4^th^, and 9^th^ months of the evaluation, showed the area under the curve (AUC) of 0.958, 0.979, and 0.910, respectively. The suggested cut-off points for different CDAI disease activity categories according to ROC curves were ≤ 4 (Remission), > 4 to ≤ 6 (low), > 6 to ≤ 18 (moderate), > 18 (high). These findings indicate that the CDAI has good concordance with DAS 28 in assessing the disease activity in RA patients, in this study sample.

## Introduction

’Treat to target’ strategy is recommended by the American College of Rheumatology (ACR) when managing Rheumatoid arthritis patients [1]. Evaluation of the disease activity is a crucial step when managing RA patients according to this strategy, as it guides clinicians in deciding the appropriate treatment, and the dose adjustments according to the therapeutic response. Furthermore, ACR has endorsed 6 composite measures of disease activity, including Disease Activity Score 28 (DAS 28) [2], Clinical Disease Activity Index (CDAI) [3] and Simplified Disease Activity Index (SDAI) [3] to measure disease activity and define remission in RA. Furthermore, the European League Against Rheumatism (EULAR) guidelines for the management of RA also recommends using such composite measures of disease activity when monitoring RA patients [4].

Among many composite measures/instruments available to assess the disease activity of RA, DAS 28 is the most widely used tool in clinical trials and patient care [2, 5, 6]. However, certain limitations deter the use of DAS 28 in some clinical settings. The four variables that are considered for DAS 28 calculation are number of tender joints (0–28), number of swollen joints (0–28), patient evaluated global health status on a visual analog scale (VAS) of 0–10, and measurement of acute phase reactants (either erythrocyte sedimentation rate (ESR) or C-reactive protein (CRP). Therefore, in the absence of measures of acute phase reactants, the calculation of disease activity using DAS 28 is restricted because ESR/CRP measurements are not readily available with the patients all the time in some clinic settings. Furthermore, the calculation of DAS 28 is complex. For instance, if ESR is considered when calculating DAS-28, the formula is DAS 28-ESR = 0.56*sqrt (tender joint count) + 0.28*sqrt (swollen joint count) + 0.70 Ln (ESR) + 0.014*global health. Thus, it warrants the use of calculators/online tools for its calculation. Therefore, regular use of DAS 28 to assess the disease activity in resource-restricted, busy clinic settings is limited.

In contrast, the assessment of disease activity in RA using CDAI is convenient and straightforward [3, 6]. The four parameters that are measured in CDAI, i.e., number of tender joints (0–28), number of swollen joints (0–28), patient global assessment of disease activity and physician global assessment of the disease activity, can easily be counted/evaluated and then summated to obtain the CDAI value. The SDAI is also similar to CDAI. However, it requires the measurement of C-reactive protein in addition to 4 parameters measured in CDAI. Thus, the need for laboratory investigations restricts the use of SDAI over CDAI. Therefore, CDAI is the simplest composite measure, compared to DAS 28 and SDAI, to assess the disease activity in RA as it can be carried out within a short period. This would also make a regular assessment of RA disease activity in some clinic settings more realistic, quick, and practical.

Studies carried out in many countries have validated CDAI in their respective populations enabling its use in those countries or populations [7–10]. To date, CDAI has not been validated in Sri Lanka despite having a higher necessity for its use. Since DAS 28 has been widely used as a gold standard composite tool for evaluating RA disease activity, it can be used as the reference tool when validating CDAI [11]. Therefore, the objective of this study was to assess the validity of CDAI compared to DAS 28, as a composite measure of disease activity assessment of RA patients in Sri Lanka.

## Materials and methods

### Patient recruitment and follow up

This prospective follow up study was carried out at the Rheumatology clinics at Teaching Hospital, Peradeniya and Rehabilitation Hospital, Digana, in the Central province of Sri Lanka. Ethical permission was obtained from the institutional ethical review committee, Faculty of Medicine, University of Peradeniya, Sri Lanka (2014/EC/65).

After obtaining the informed written consent, newly diagnosed RA patients, who fulfilled the ACR/EULAR 2010 criteria, were recruited to the study using consecutive sampling method [12]. The study was conducted between August 2014 to May 2017. Data on patient demographics and disease characteristics were gathered by history taking and physical examination. A total of 28 bilateral joints, i.e., shoulder, elbow, wrist, metacarpophalangeal, proximal interphalangeal, and knee joints, were assessed for tenderness and swelling for the subsequent calculation of disease activity. DAS 28 was calculated using an online DAS 28 calculator (http://www.das-score.nl/das28/en) as the reference method of disease activity assessment. CDAI was calculated by summating the tender joint count, swollen joint count, patient evaluation of disease activity and the disease activity evaluated by the physician. This was the baseline or 0-month evaluation. After the baseline (0 months) evaluation, the patients were evaluated again for the disease activity using the DAS 28 and CDAI at 4 and 9 months during their routine and monthly visits to the clinics.

Disease activity, as measured by DAS 28 and CDAI, at these 3 stages of evaluation, were used to validate CDAI compared to DAS 28. The cut off points for DAS 28 for evaluation of disease activity were; >5.1(high), ≥3.2 to ≤5.1(moderate), ≥2.6 to <3.2 (low), and <2.6 (remission) [5]. Cut off points with respect to the CDAI were; >22 (high), >10.0 to 22.0 (moderate), >2.8 to 10.0 (low) and ≤2.8 (remission) [3].

### Statistical analysis

Data analysis was carried out using either parametric or non-parametric statistical tests, depending on the normality of data distribution. Internal consistency reliability of CDAI was evaluated by Cronbach’s alpha coefficient. Possible values of Cronbach’s alpha ranged from 0 (no internal consistency) to 1 (identical results). Convergent validity was examined by Spearman’s correlation coefficient analysis and inter-rater agreement (Kappa statistics). Spearman’s correlation coefficient was assessed by correlating the scores of the CDAI with that of DAS 28 at three stages of patient evaluation. Patients were grouped into 4 disease activity groups (remission, low, moderate, and high disease activity) based on CDAI and DAS 28 cut-off points to assess the inter-rater agreement, and the weighted kappa was calculated. Receiver operator characteristic (ROC) curves were constructed to assess the discriminant validity of CDAI as a tool that discriminates disease activity of RA as categorized by the various cut off points (5.1, 3.2, 2.6) of DAS 28. These curves plotted the true positive rate against the false-positive rate for the different possible cut off points of the CDAI. Each point on the ROC curve indicates a CDAI value, which has a sensitivity/specificity pair corresponding to a particular decision threshold. CDAI cut-off points were decided based on the Youden’s J statistic [13]. In the ROC curve plot, J is represented by the maximum vertical distance from the ROC curve to the diagonal line (50% sensitivity, 50% specificity). While the ROC curve of a worthless test falls on the diagonal line, a test with perfect discrimination (without overlap in the two distributions) has a ROC curve that passes through the upper left corner (100% sensitivity, 100% specificity). Therefore, closer the ROC curve is to the upper left corner, the higher the overall accuracy of the test [14]. Three ROC curves were constructed corresponding to 3-time points of disease activity evaluation.

Statistical analysis and data visualization were conducted using MedCalc for Windows, version 20.115 (MedCalc Software, Ostend, Belgium) and R (version 4.0.4) [15]. P < 0.05 was considered statistically significant.

### Results

One hundred and three patients were recruited to the study and evaluated at the first visit. Most of the patients were women, and the mean age was approximately 49 years (Table 1). Nearly two-thirds of the studied patient group had not completed secondary education. Of them, only 86 patients at 4 months and 77 patients at 9 months were available in the clinics for evaluation. The rest of the patients had discontinued the treatment or lost during the follow-up.

**Table 1:** Socio-demographic characteristics of the patient cohort (n = 103).

	n (%)
Gender ratio (women: men)	6:1
Mean ± SD of age (years) (mean ± SD)	49±11
Age groups (years)	
20–35	13 (12.6)
36–50	39 (37.9)
51–65	47 (45.6)
66–80	4 (3.9)
Ethnicity	
Sinhalese	84 (81.6)
Tamils	9 (8.7)
Moors	10 (9.7)
Family history of RA	17 (16.35)
Education	
Incomplete primary education	2 (1.9)
Complete primary education	12 (11.7)
Incomplete secondary education	64 (62.1)
Complete secondary education	20 (19.4)
Complete tertiary education	5 (4.9)

RA- rheumatoid arthritis, SD-standard deviation

Of the 103 patients recruited, which was based on the 2010 ACR/EULAR classification criteria, the majority had a polyarticular pattern of joint involvement (77.6%). The mean tender joint count was 8.40, and the mean swollen joint count was 4.61 (Table 2). Considering the symptom duration at the baseline evaluation, 69 patients were classified as early RA (disease duration less than ≤6 months) and 34 patients as established RA (disease duration > 6 months). Rheumatoid factor positivity was seen in 76% of the cases.

**Table 2:** Disease characteristics of RA patient at their initial presentation.

Variable	Value
Mean disease duration (months) (mean ± SD)	6.3±4.4
Joint involvement	
Oligoarticular (2–4) joints—n (%)	22 (21.3)
Polyarticular (>4) joints—n (%)	81 (78.7)
Duration of morning stiffness—n (%)	
> 2 h	14 (13.7)
>1 h	43 (41.7)
< ½- 1 h	40 (38.8)
< ½ h	6 (5.8)
Swollen joint count (mean ± SD)	4.6 ± 3.4
Tender joint count (mean ± SD)	8.4 ± 4.9
Joint pain- VAS (0–10) (mean ± SD)	5.6±1.9
DAS 28 (mean ± SD)	5.7 ±1.0
CDAI (mean ± SD)	24.1±9.8
Rheumatoid factor- (% positivity)	79 (76.7)
ESR mm 1^st^ hour (mean ± SD)	57.9 ± 24.5

SD–standard deviation, VAS- visual analog scale, DAS 28 –disease activity score 28, CDAI- clinical disease activity index.

Patients were given either monotherapy or combined therapy of disease-modifying anti-rheumatic drugs (DMARDs), methotrexate (62%), sulfasalazine (25%), methotrexate and sulfasalazine (12%), and methotrexate and hydroxychloroquine (1%). In addition to DMARDs, all patients were on non-steroidal anti-inflammatory drugs (NSAIDs), and some patients were on short courses of prednisolone.

**Fig 1:**
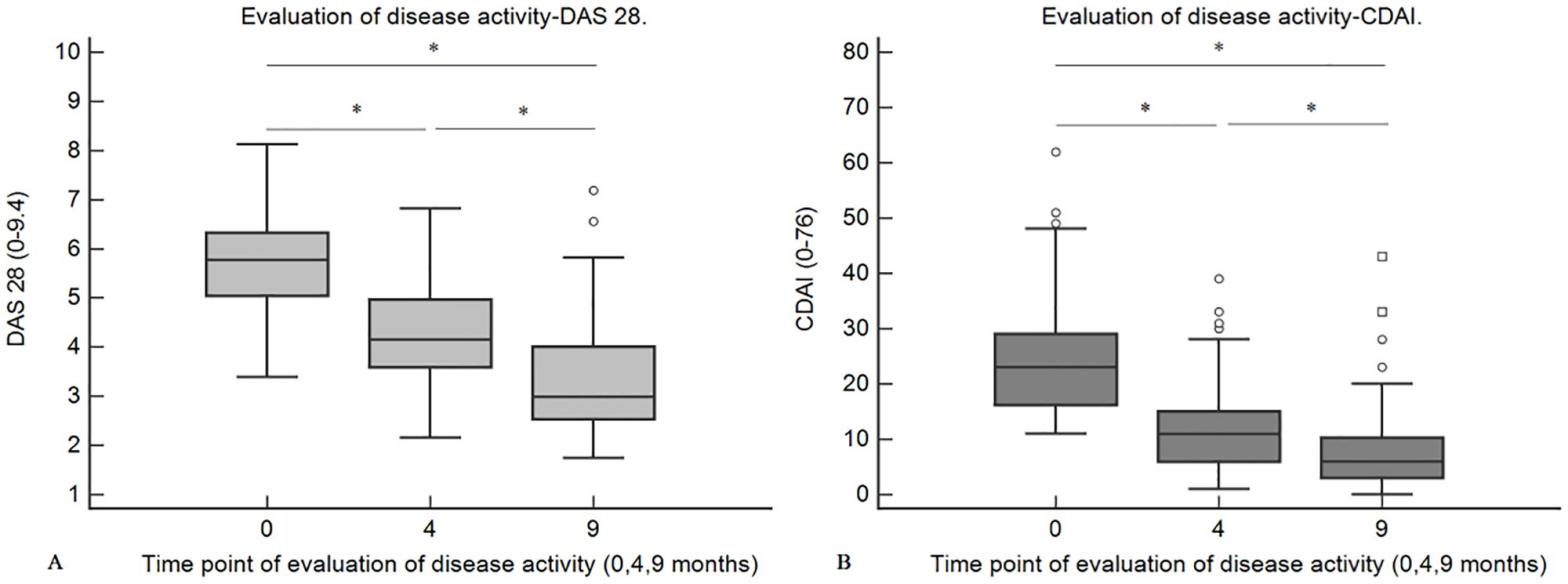
Box and whisker plots illustrating the changes in the disease activity in the patient cohort as assessed by CDAI (a) and DAS 28 (b). The median disease activities at different time points were compared by the Kruskal-Wallis test (with Dunn’s post-hoc analysis). The drops in the median disease activity down the timeline were statistically significant (p < 0.05). Asterix indicate that the difference between groups is statistically significant. DAS 28 –disease activity score 28, CDAI- clinical disease activity index, 0, 4, and 9 in the x-axis indicate the time point of evaluation (0 –baseline, 4–4 months, and 9–9 months).

The disease activity of each of the patients was evaluated using the two composite measures, i.e., DAS28 and CDAI. Among the patients who regularly followed up in the clinic, it was observed that the disease activity, as assessed by both CDAI and DAS 28, had reduced gradually from the initial presentation to the 9 months review (Fig 1). The drops in the disease activity for both CDAI and DAS 28 were statistically significant (Kruskal-Wallis test with post-hoc analysis (Dunn), (p < 0.05)).

The reliability of CDAI was tested with the internal consistency reliability (Cronbach’s α) test, and the validity was tested with correlation, inter-rater agreement, and ROCs. For testing the validity of CDAI, DAS 28 was used as the reference.

Internal consistency reliability analysis of 4 items of the CDAI (tender joint count, swollen joint count, patient evaluation of disease activity, and physician evaluation of disease activity) was carried out. Cronbach’s α indicated that the composite disease activity measure CDAI has good reliability, α = 0.846, according to the reference values described by George D. et al (2003) [16].

**Fig 2:**
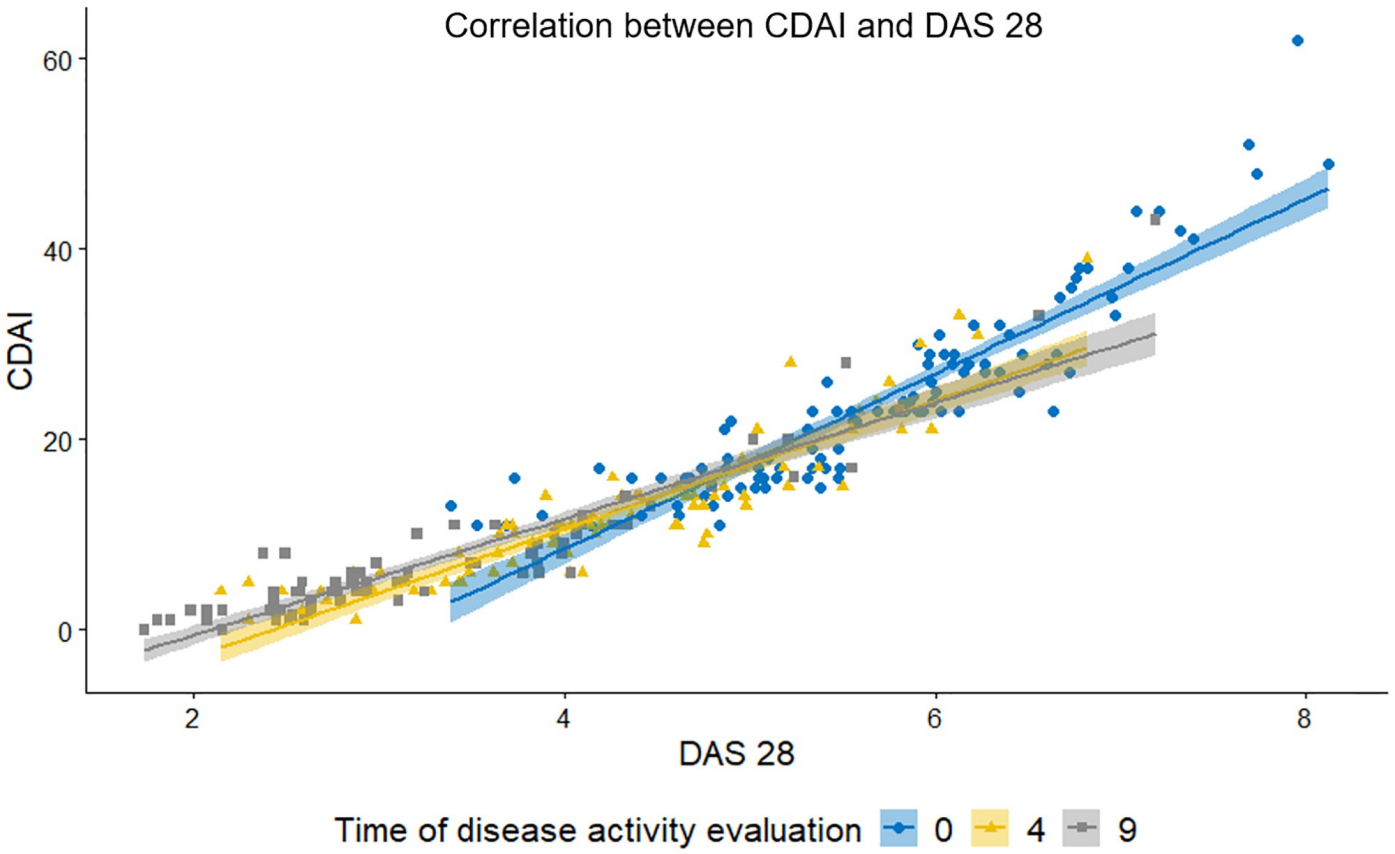
Scatter plot illustrating linear positive correlation between DAS 28 and CDAI assessed at 3 time points (0 = 0 months, 4 = 4 months, 9 = 9 months). At all three time points of evaluation, CDAI was strongly correlated with DAS 28; ρ = 0.9357 at 0 months (p<0.05), ρ = 0.9354 at 4 months (p<0.05), ρ = 0.9106 at 9 months (p<0.05). CDAI–clinical disease activity, DAS 28- disease activity score 28.

Correlations of the disease activity as assessed by CDAI and DAS 28 were calculated with the Spearman correlation coefficient. At all three time points of evaluation, the 2 variables showed a very strong correlation (ρ > 0.8) [17]. Fig 2 illustrates the correlation of the disease activity as assessed by CDAI and DAS 28 at baseline (0 months), 4 months, and 9 months.

According to the assessment of the inter-rater agreement, by weighted Kappa statistics, moderate-good agreements were observed between CDAI and DAS 28 based disease activity categorization (Table 3). The strength of agreements was good at baseline and 9 months, whereas moderate at 4 months, according to the reference values described by Altman, D. (1991) [18].

**Table 3:** Inter-rater agreement between CDAI and DAS 28 at 3-time points of disease activity assessment.

Stage of evaluation	Weighted Kappa^a^	Standard error	95% CI
0 months (baseline)	0.660	0.080	0.503–0.817
4 months	0.519	0.072	0.377–0.660
9 months	0.741	0.053	0.636–0.845

^a^Linear weights. Strength of agreement based on value of K: < 0.20 (poor), 0.21–0.40 (fair), 0.41–0.60 (moderate), 0.61–0.80 (good), 0.81–1.00 (very good) [18].

Three separate ROC curves were constructed, by plotting the true-positive rate against the false-positive rate of CDAI, to evaluate the overall usefulness of CDAI, and to identify the best cut-off points that correspond to DAS 28 cut off points.

**Fig 3:**
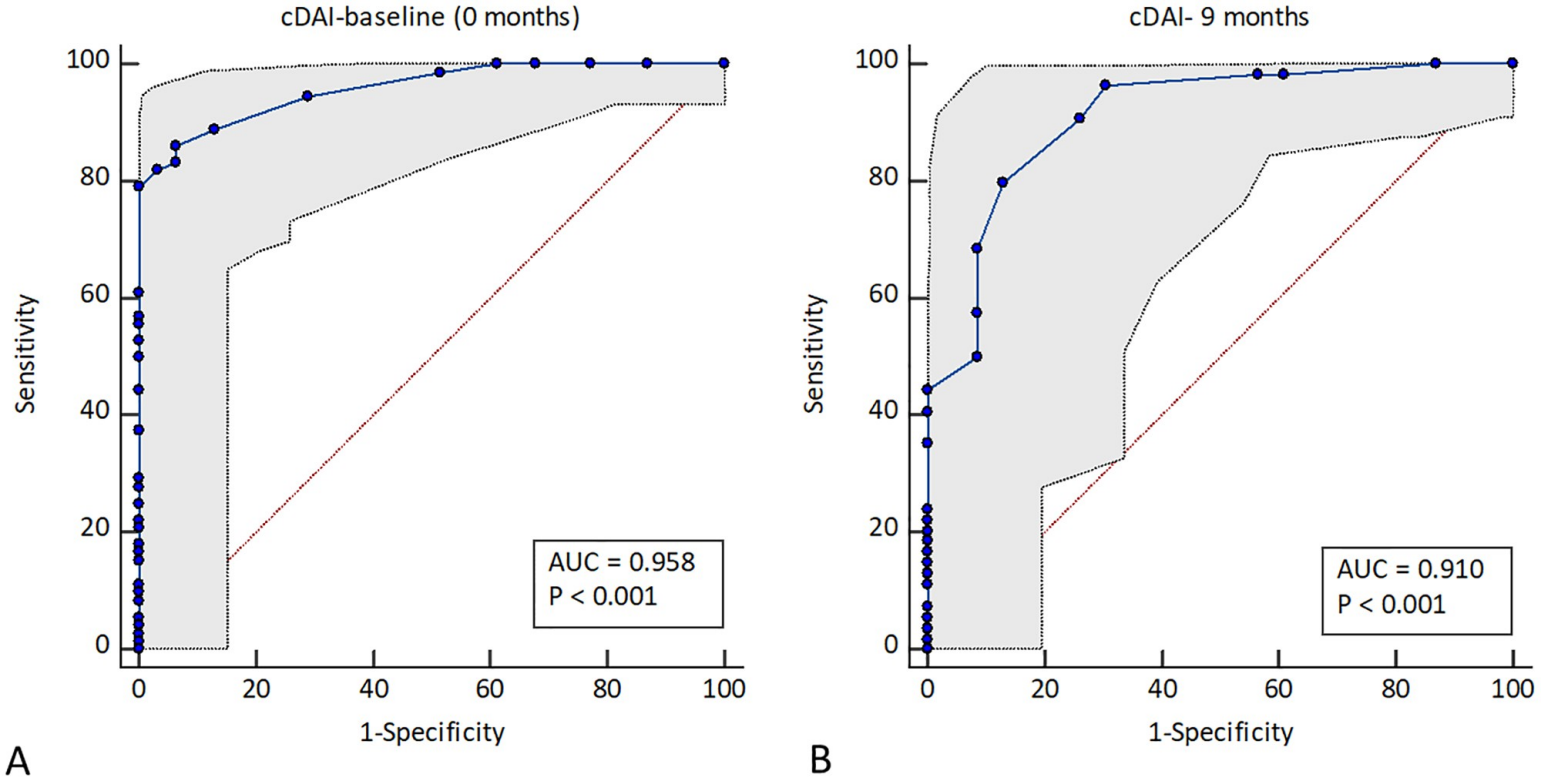
ROC curves. **A**. ROC curve illustrating the sensitivity and 1-specificity for different values of CDAI corresponding to a DAS28 cut off value of 5.1, based on the disease activity evaluation at the first visit (0 months). Circles on the ROC curve corresponds to different criterion values of CDAI, **B**. ROC curve illustrating the sensitivity and 1-specificity for different values of CDAI corresponding to a DAS28 >2.6, based on the disease activity evaluation at 9 months. Optimum CDAI cut-off point were decided based on the Youden’s J statistic. Circles on the ROC curve corresponds to different criterion values of CDAI. CDAI–clinical disease activity, DAS 28- disease activity score 28.

Since most patients had high disease activity (69%) at baseline (0 months) evaluation (DAS 28 >5.1), a ROC curve was constructed to determine the sensitivity and specificity of different values of CDAI which would differentiate between a DAS 28 value > 5.1 or <5.1. The best combination of sensitivity (86.11%) and specificity (93.55%) was provided by a CDAI value of 18 (Fig 3A). The AUC was 0.958 (p < 0.05). When patients were evaluated at 4 months, the majority of the patients had moderate disease activity (62%) (DAS 28 of 3.6–5.1). Hence, the second ROC curve was constructed to identify the sensitivity and specificity of different values of CDAI, which would differentiate between a DAS 28 value >3.2 or < 3.2 (S1 Fig). The best combination of sensitivity (88.41%) and specificity (100%) was provided by a CDAI value of 6. The AUC was 0.979 (p < 0.05). During the patient evaluation for disease activity at 9 months, most patients (53%) had low disease activity or remission. Therefore, based on the disease activity assessment at 9 months using DAS 28, the third ROC curve was constructed to decide the sensitivity and specificity of different values of CDAI, which differentiate between a DAS 28 value >2.6 or < 2.6 (Fig 3B). The best combination of sensitivity (79.6%) and specificity (86.96%) was provided by a CDAI value of 4. The AUC was 0.910 (p < 0.05).

## Discussion

The use of composite measures, such as CDAI, to assess the disease activity in RA is vital both in regular clinical practice as well as in clinical trials. Even though most of these instruments are intended to measure disease activity as accurately as possible, these measures have validated only in specific populations. Thus, it is needed to validate these measures in the desired population before it is being used. However, in an era of rapidly evolving medical diagnostics, evaluation of the disease activity of RA by clinical tools such as CDAI, that do not depend on laboratory measures, may not be impressive. One might argue that ignoring inflammatory markers such as ESR or CRP is disadvantageous when assessing the disease activity. However, it is shown that there is no major contribution by inflammatory markers to the total value of composite measures of RA disease activity such as DAS28 [3]. Furthermore, in a study carried out by Kay et al., among 9,135 with active RA, only 42% of patients had elevated levels of ESR or CRP [19]. Furthermore, it is observed that evaluation of disease activity using a composite measure such as DAS 28 is not routinely practised. From the patient’s point of view, even though they are supposed to get such investigations done before the clinic visit, some of the patients seem to be non-compliant. All these factors have limited the methodical evaluation of disease activity using DAS 28. Therefore, the decision making on therapeutic adjustments has become a challenge and implementing the "treat to target" strategy seems unrealistic in such situations. In this background, the use of CDAI seems to be useful as it does not depend on any laboratory investigation and the simplicity of its calculation. However, CDAI had not been validated previously in a Sri Lankan cohort of RA patients.

The validation of CDAI was conducted, recruiting a cohort of newly diagnosed RA patients from two Rheumatology clinics in Sri Lanka. Key features of the recruited patient sample at the time of diagnosis were short symptom duration at presentation (6.33 months), middle-age of disease onset (mean 49 years), remarkable female preponderance (6:1), polyarticular involvement (83.4%) involvement of more hand joints and higher mean disease activity at baseline (mean DAS 28 of 5.69).

Disease activity in this patient cohort, as assessed by DAS 28 and CDAI, gradually decreased during the follow-up. This is possibly attributed to the early diagnosis of RA using 2010 ACR//EULAR criteria at Rheumatology clinics during recruiting patients to the study [12]. Moreover, early commencement of DMARDs and higher literacy among these patients leading to better adherence to treatment may have attributed to such a better therapeutic outcome.

CDAI was evaluated using different statistical methods, including internal consistency reliability, convergent validity, and discriminant validity. A good internal consistency reliability (Cronbach’s α - 0.846) for CDAI, in which the correlation between the 4 items in CDAI were assessed, reflects that the 4 items of CDAI indicate similar information. Convergent validity of CDAI was also found to be good, as shown by correlation and weighted Kappa statistics. Correlation analysis of the disease activity measured using CDAI and DAS28 at 3-time points of evaluations revealed a ’very strong’ correlation as Spearman’s ρ found to be between 0.8–1.0 in all three points. This finding is parallel with that of other CDAI validation studies carried out in RA patient populations in India and Morocco [8, 10]. Comparison of the proportion of patients under each disease activity category, according to CDAI and DAS28, revealed a good inter-rater agreement at baseline and 9 months, and moderate inter-rater agreement at 4 months (Weighted Kappa statistics). Studies carried out in other populations have demonstrated good inter-rater reliability between CDAI and DAS 28 measures [8, 10].

The ROC curves proved the discriminant validity of the CDAI. Based on our data, plotted 3 ROC curves fell closer to the upper left corner. Therefore, the AUCs in all three ROC curves were above 0.9, indicating an outstanding discriminant validity of CDAI compared to DAS 28. Moreover, such a higher AUC indicates the clinical use of CDAI.

Based on the constructed ROC curves, cut-off values were derived for different categories of disease activity. Table 4 illustrates the proposed cut-off values for CDAI in our study with cut-off values derived from those of the original validation study of CDAI by Aletaha D. et al (2005) [20] and another comparable study by Arya V. et al (2007) [21]. It is apparent that the newer cut-off values derived from the ROC curves of the current study have some deviations from the corresponding cut-off values of 2 other studies. The reason for such discrepancy is not apparent. If these proposed cut-off points were applied to the CDAI measures of the current study, more number of patients would have been classified as having high disease activity and remission compared to the use of original cut-off points.

**Table 4:** CDAI cut-off values derived from different studies.

	Aletaha et al. (2005)	Arya et al. (2007)	Current study
Remission	≤ 2.8	≤2.2	≤ 4
Low Disease activity	>2.8 ≤ 10	> 2.8 ≤ 5	>4 ≤ 6
Moderate Disease activity	>10 ≤ 22	> 5≤ 21	>6 ≤ 18
High disease activity	>22	> 21	> 18

Many of the other population-based studies for CDAI validation were cross-sectional studies. In contrast and comparison, this study was a prospective follow up study where disease activity was assessed thrice in most of the patients during the follow-up. The purpose of this validation is to see how well CDAI can perform in assessing the disease activity in a cohort of Sri Lankan RA patients. As CDAI is evaluated compared to DAS 28, it is not possible to evaluate the better composite measure. Overall, as per the validation methods used in this study, CDAI seems to perform similar to DAS 28 in evaluating the disease activity in RA.

Even though formal evaluation of disease activity in RA is of paramount importance to follow the `treat to target`strategy, it is questionable how many clinicians and clinics practically follow this approach regularly. A study has shown that most of the patients in most clinics are not subjected to formal joint evaluation [22]. Instead, many rely on single measures of disease activity, such as acute phase reactants. However, composite measures, such as CDAI, integrate various aspects/dimensions of the disease and thus provide a holistic picture of the disease activity in RA. As CDAI performs similar to DAS 28 in this study, it is needed to encourage the assessment of disease activity of RA with CDAI as its calculation also is easy to perform.

Interobserver variations that may impact the results when multiple evaluators examine patients have been overcome in the current study as only a single investigator evaluated the joints of all patients throughout the study, which is one of the major strengths of this study in the comparative literature. However, the study can be made statistically stronger by recruiting a larger number of patients from multiple centers/ clinics.

## Conclusion

It was observed that CDAI has a good concordance with DAS 28 in assessing the disease activity in RA patients. Also, CDAI is easy to use in busy and routine clinics which doesn’t necessitate the investigations such as CRP or ESR for which patients are sometimes non-compliant in bringing to the clinics during their follow-up visits. Thus, the use of CDAI would facilitate the physicians for "Treat to Target" enabling better management of RA.

## Supporting information

S1 Fig ROC curve illustrating the sensitivity and 1-specificity for different values of CDAI corresponding to a DAS28 cut off value of 3.2, based on the disease activity evaluation at 4 months. (TIF)

S1 File Raw data. (XLSX)
