# Supplementary figures and images for: Validity of clinical disease activity index (CDAI) to evaluate the disease activity of rheumatoid arthritis patients in Sri Lanka: A prospective follow up study based on newly diagnosed patients
Source: PLoS One. 2022 Nov 29;17(11):e0278285. doi: 10.1371/journal.pone.0278285 (PMC9707768; doi:10.1371/journal.pone.0278285)

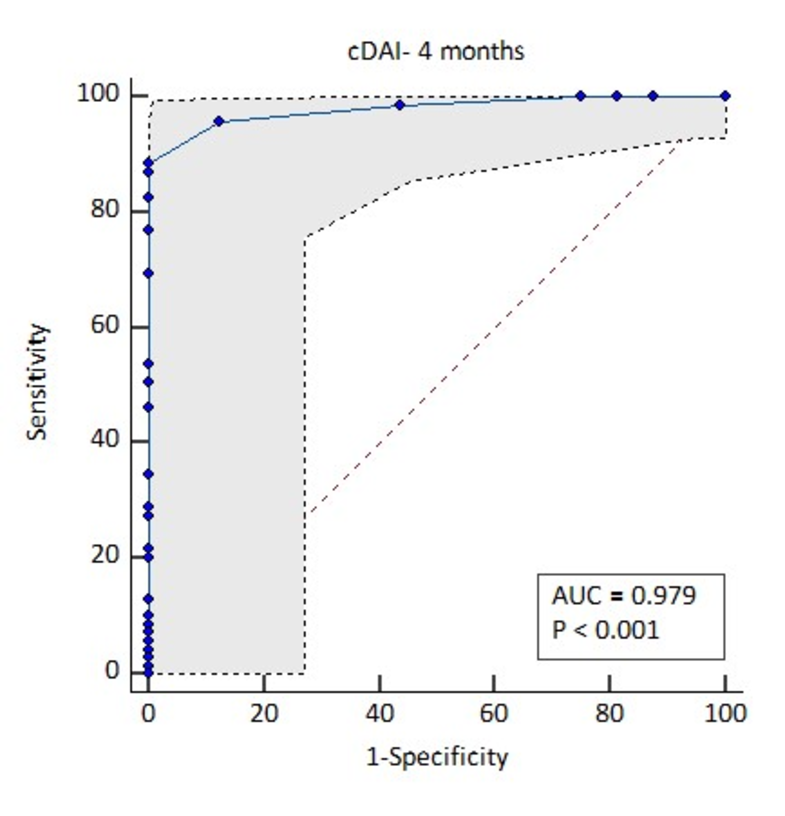

Supplement: S1 Fig — (TIF) [file pone.0278285.s001.tif]
